# Supplementary material for: Broadband transparent optical phase change materials for high-performance nonvolatile photonics
Source: Nat Commun. 2019 Sep 30;10:4279. doi: 10.1038/s41467-019-12196-4 (PMC6768866; doi:10.1038/s41467-019-12196-4)
Supplement: Supplementary file 1 — Supplementary Information [file 41467_2019_12196_MOESM1_ESM.pdf]

Supplementary Information for

"Broadband Transparent Optical Phase Change Materials for High-Performance Nonvolatile Photonics"

Zhang et al.

Supplementary Note 1: DFT atomic model construction and transition kinetics inferred from static energy analysis

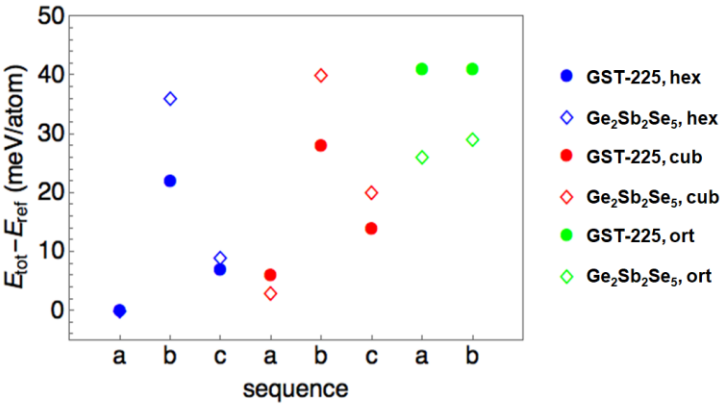

Supplementary Figure 1. Cohesive energies of GST-225 and $Ge_2Sb_2Se_5$ with various stacking sequences of the hexagonal, cubic, and orthorhombic phases relative to that of sequence (a) of the hexagonal phase. The sequences are defined as: (a) -Te-Ge-Te-Sb-Te-Te-Sb-Te-Ge-; (b) -Te-Sb-Te-Ge-Te-Te-Ge-Te-Sb-; and (c) Te-Sb/Ge-Te-Sb/Ge-Te-Te-Sb/Ge-Te-Sb/Ge-. Legend: (hex) hexagonal; (cub)

We start by constructing atomic models of three phases known to emerge in the $Ge_2Sb_2Se_xTe_{5-x}$ system: hexagonal, cubic, and orthorhombic[1]. Given the structural complexity in quaternary GSST, it is computationally demanding to perform brute-force searches across the entire structural space. Instead, we deduced the possible configurations based on general rules unveiled by looking into the upper and lower limits of Se substitution.

To gain insights into the impact of Se substitution on lattice structure and cohesive energy of the different phases, we constructed hexagonal, cubic, and orthorhombic lattice models for the two end-point compositions, GST-225 and $Ge_2Sb_2Se_5$, with the same atomic arrangement. For each lattice type, the relative stability of different atomic stacking sequences (i.e. the same arrangement while exchanging atom types) were also computed via DFT to identify the most energetically favorable structure. The computed results are summarized in Supplementary Table 1. The results indicate the same trend of phase stability for GST-225 and $Ge_2Sb_2Se_5$ (Supplementary Figure 1). Data in Supplementary Table 1 also suggest that Se substitution mainly induces isotropic contraction of the unit cells with little distortion in all three structures. The finding implies that the energetically favorable stacking sequences identified in the two end-point compositions can be equally applied to the intermediate GSST compounds with partial Se substitution.

Supplementary Table 1. Cohesive energies and lattice constants of hexagonal, cubic, and orthorhombic phases of GST-225 and $Ge_2Sb_2Se_5$. E_b/atom: cohesive energy on a per atom basis; *a*, *b*, and *c*: lattice constants.

		GST-225	$Ge_2Sb_2Se_5$	Percentage change as a result of Se substitution
Hexagonal	E_b/atom (eV)	3.33	3.59	7.6
	a (Å)	4.24	3.99	-5.8
	c (Å)	16.87	15.91	-5.7
Cubic	E_b/atom (eV)	3.33	3.58	7.7
	a (Å)	4.22	3.99	-5.6
	c (Å)	51.76	48.75	-5.8
Orthorhombic	E_b/atom (eV)	3.29	3.56	8.2
	a (Å)	25.19	23.81	-5.5

	b (Å)	4.23	3.98	-5.8
	c (Å)	20.45	19.27	-5.7

Supplementary Table 2. Cohesive energies of hexagonal GSS1T4 in sequence (a) with various Se atom distributions illustrated in Supplementary Figure 2. E_b/atom: cohesive energy on a per atom basis

Configuration	E_b/atom (eV)
cf1	3.377
cf2	3.383
cf3	3.373
cf4	3.374
cf5	3.371
cf6	3.373
cf7	3.379
cf8	3.378

We then considered 20% Se substitution in various configurations to analyze relevant chemical features. Supplementary Table 2 summarizes the DFT-computed cohesive energies of prototype systems of hexagonal GSS1T4 in sequence (a) with various Se atom distributions illustrated in Supplementary Figure 2. The information is used to extract the dependence of cohesive energies on feature chemical components. By comparing the relative stability of the different structures, we identify that the bonding energies obey the following trend in decreasing order: Se-Sb > Se-Ge > Se-Se. The conclusion is consistent with experimental findings reported for amorphous $Ge_2Sb_2Se_5$, where Se-Se pairs are rarely present, Se atoms are surrounded by Ge or Sb atoms, and Ge and Sb atoms tend to bond to Se than to Ge or Sb atoms[2]. The most energetically favorable structure (cf2) contains the strongest Se-Sb bonds and exhibits the tendency of Se atom aggregation in-plane to eliminate stress. The high energies of the cf3/cf4/cf5/cf6 structures unveil the large energy penalty to form under-coordinated Se at the weakly bound Te-Te double layer.

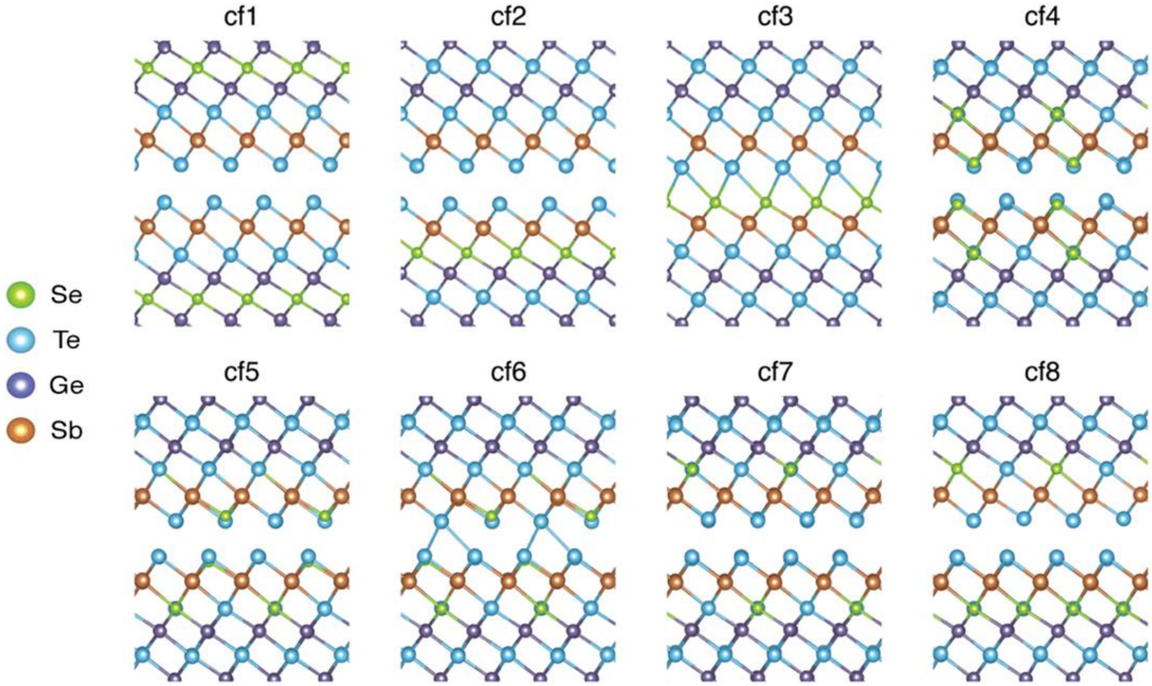

Supplementary Figure 2. Atomic configurations of hexagonal GSS1T4 in sequence (a) evaluated in this study

Based on the above structural analyses of GST-225, $Ge_2Sb_2Se_5$, and structural variants of GSS1T4, we derived the energetically favorable structures for hexagonal and orthorhombic GSST crystals as shown in Supplementary Figure 3 and 4, respectively. For hexagonal $Ge_2Sb_2Se_xTe_{5-x}$ with x = 0, 1, 2, 3 and 5, the structures are deterministic as Se atoms tend to aggregate at the same plane away from the Te-Te double layers. In contrast, for hexagonal GSS4T1 (x = 4), the Se atoms within the Te-Te double layer can both sit inside a plane and mix with Te atoms, as seen from the similar cohesive energies (3.537, 3.536, 3.537 eV/atom) computed for the three prototype configurations (cf1, cf2 and cf3 in Supplementary Figure 3). Regarding the orthorhombic phase, the Se atoms substitute the 6-fold coordinated Te inside the atomic blocks at low Se concentration, while filling the remaining interstitial sites at high Se concentration ($x \geq 3$).

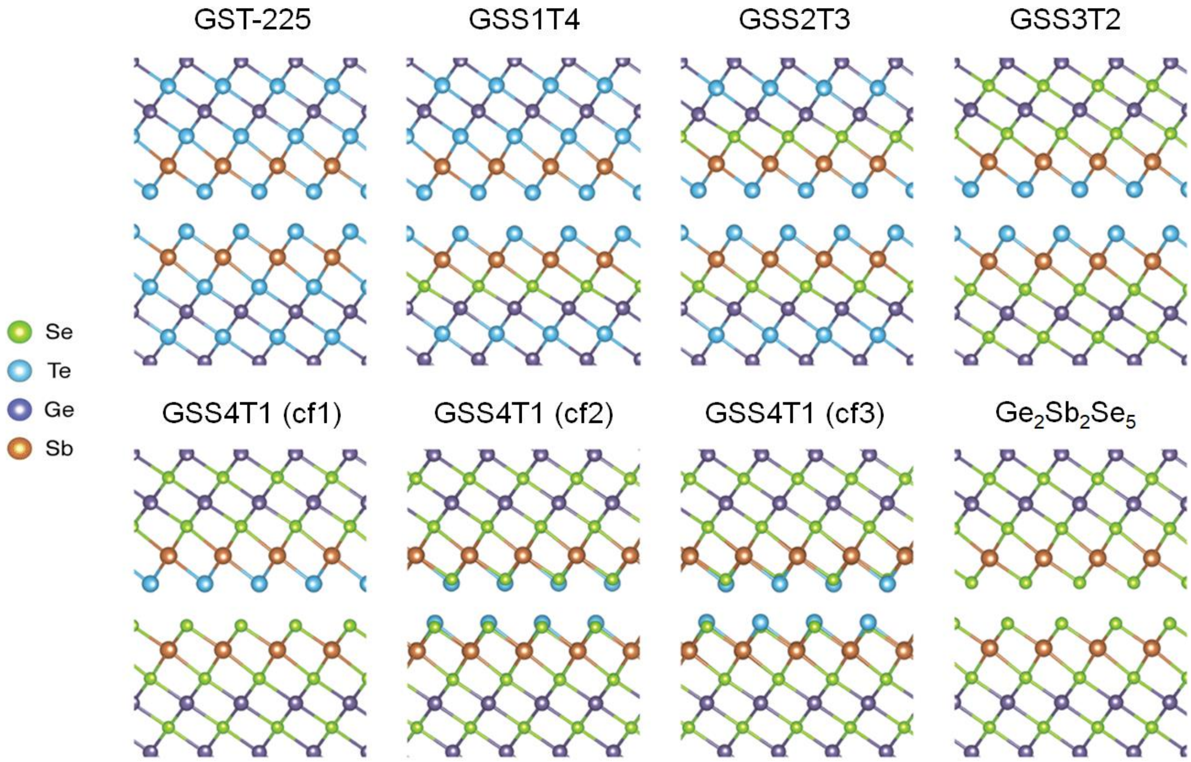

Supplementary Figure 3. Atomic configurations of hexagonal $Ge_2Sb_2Se_xTe_{5-x}$

The structural model further provides qualitative insight into the phase transition kinetics of the alloys. Similar to GST-225 whose rapid phase change kinetics necessitates a non-diffusion-controlled process[3,4], phase transition in GSST is also associated with a combination of atom block movement and local distortion. However, atomic block movement in GSST is more difficult to occur compared to GST-225 due to the presence of relatively strong Te-Se bonds at the interfacial layers (bond energies of Te-Te, Te-Se, Se-Se, and Se-Ge are 33.0, 40.6, 44.0, and 49.0 kcal/mol, respectively[5]). When sufficiently high temperature for Te-Se bond dissociation is reached to unlock atomic block motion, the Te-Te bonds have already broken, likely accompanied by substantial surface reconstruction. Such atomic arrangement perturbation suppresses sliding along the weakly bound Te/Se-Te/Se double layer and formation of vacancy layers essential to the transition towards the metastable cubic phase. Consequently, the temperature window for the metastable phase formation is expected to decrease with increasing Se substitution.

Unlike the cubic phase, the orthorhombic phase's relative stability substantially increases with Se concentration when $x \geq 3$. For GST-225, the orthorhombic phase introduces under-coordinated atoms at the interstitial sites and is energetically unfavorable. At low Se concentration, the Se atoms substitute 6-fold coordinated Te atoms with little impact on the relative stability of the orthorhombic phase. When $x \geq 3$, Se atoms start to fill the interstitial sites with more Se-Ge/Sb bonds formed within the orthorhombic phase than in the hexagonal phase, which stabilizes the orthorhombic phase. The orthorhombic structure is further stabilized at full Se substitution owing to the partial release of geometric restriction imposed by the Te atoms and the consequential reduction of Se-Ge/Sb bond lengths. In addition, pinning by Se-Ge bonds at the interstitial sites impedes atomic block motion, pointing to slow crystallization kinetics in $Ge_2Sb_2Se_5$.

We note that the lower stability of the orthorhombic phase of $Ge_2Sb_2Se_5$ compared to those of other phases predicted by DFT calculations is inconsistent with experimental observation. Such discrepancy may arise from two reasons: 1) the intrinsic imperfections of exchange-correlation functional to describe the dependence of energy on density gradient and to address the derivative discontinuity of Kohn-Sham potential make standard DFT approaches inefficient to predict the relative stability of various phases with distinct bonding characters and similar cohesive energies; 2) the DFT simulations determine the structures and energies at 0 K, while lattice phonon vibrational analysis is demanded to predict finite-temperature phase stability.

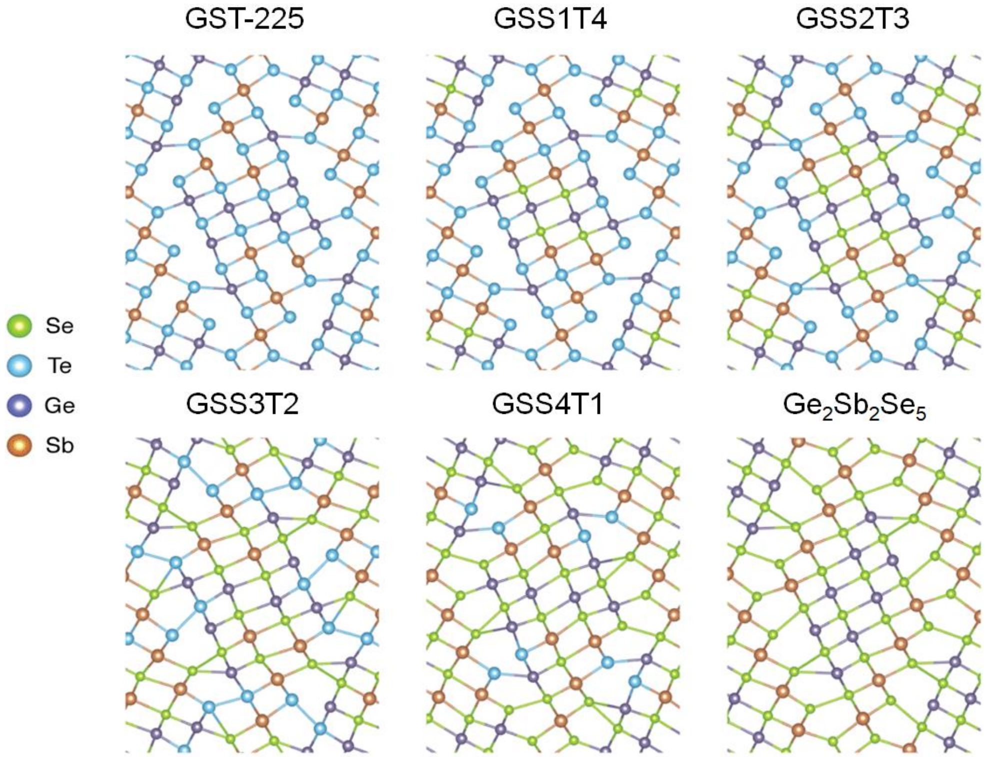

Supplementary Figure 4. Atomic configurations of orthorhombic $Ge_2Sb_2Se_xTe_{5-x}$

While static energy analysis is efficient to predict relative stability as demonstrated by previous comparison between theoretical and experimental data[6], quantitative description of crystallization kinetics requires *ab initio* molecular-dynamics (AIMD) simulations tracing bond variation in the nanosecond timescale, which is beyond the scope of this study[7-10]. Nevertheless, prior work based on AIMD simulations revealed that the Ge-Te core network is responsible for the rapid phase switching in GST materials[9], and that the presence of minority bond types in $Ag_{3.5}In_{3.8}Sb_{75.0}Te_{17.7}$ (AIST) substantially reduces the effective transition barriers[10], our postulate based on the strength of Te-Te and Te-Ge bonds modified by the Se dopant likely provides a valid qualitative trend.

Supplementary Note 2: Electronic structures of $Ge_2Sb_2Se_xTe_{5-x}$

We examined the dependence of electronic structure on chemical composition and phase by considering GST-225 and $Ge_2Sb_2Se_5$ crystals of hexagonal and orthorhombic phases. The results suggest that for both phases, Se substitutions shift part of the states near the band edge deeper into the valence/conduction bands as illustrated in Supplementary Figure 5. The band gap substantially widens with the transition from hexagonal to orthorhombic phase regardless of the chemical composition. Thus, the distinct behaviors observed in the $Ge_2Sb_2Se_xTe_{5-x}$ alloys should be ascribed to modifications of both the phase and chemical composition.

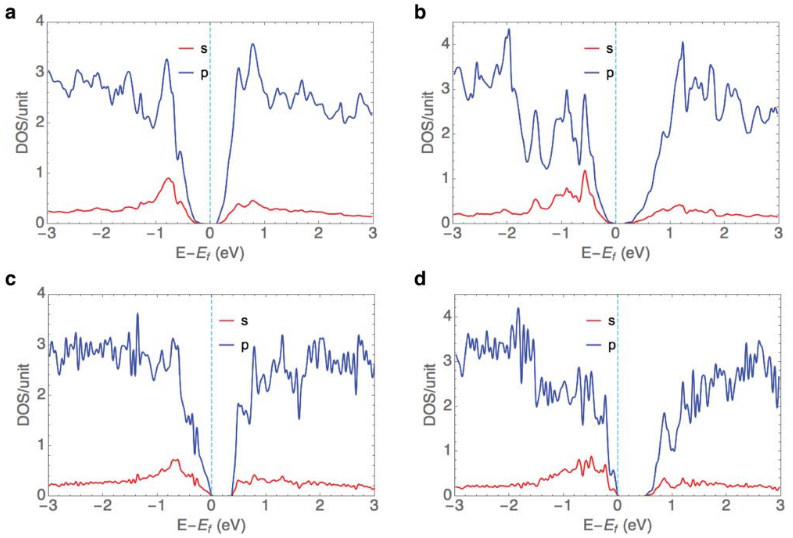

Supplementary Figure 5. Projected density of states (PDOS) on orbitals for (a, c) GST-225 and (b, d) $Ge_2Sb_2Se_5$ crystals of (a, b) hexagonal and (c, d) orthorhombic phases

As mentioned in Supplementary Note 1, hexagonal GSS4T1 exhibits atomic disorder within the Te/Se-Te/Se double layers. The impact of such atomic disorder on electronic structure was investigated by comparing the PDOS of configurations cf1 and cf3. Because of the similar characteristics of Te and Se atoms, random atomic mixing of Se and Te only slightly modifies the density of states as shown in Supplementary Figure 6.

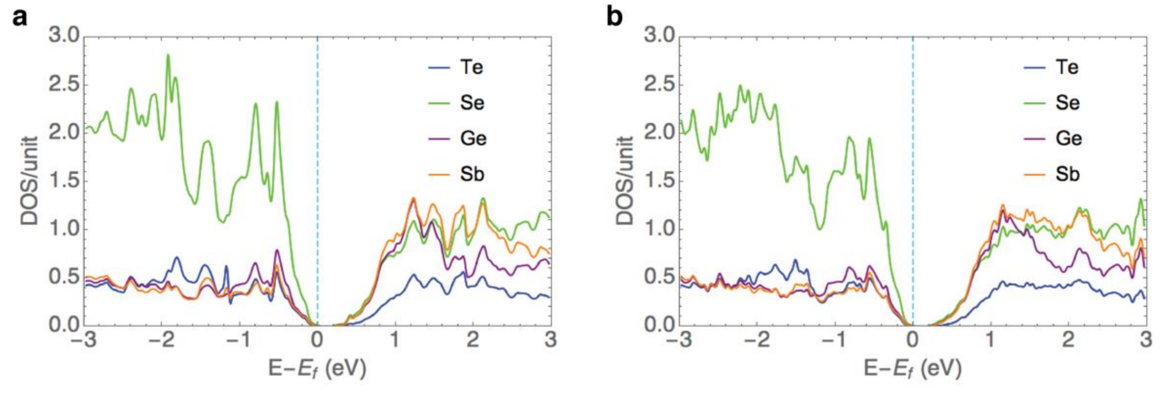

Supplementary Figure 6. PDOS on atoms for GSS4T1 (a) without and (b) with atomic disorder within the Te/Se-Te/Se double layers

Supplementary Note 3: X-ray diffraction analysis of $Ge_2Sb_2Se_xTe_{5-x}$

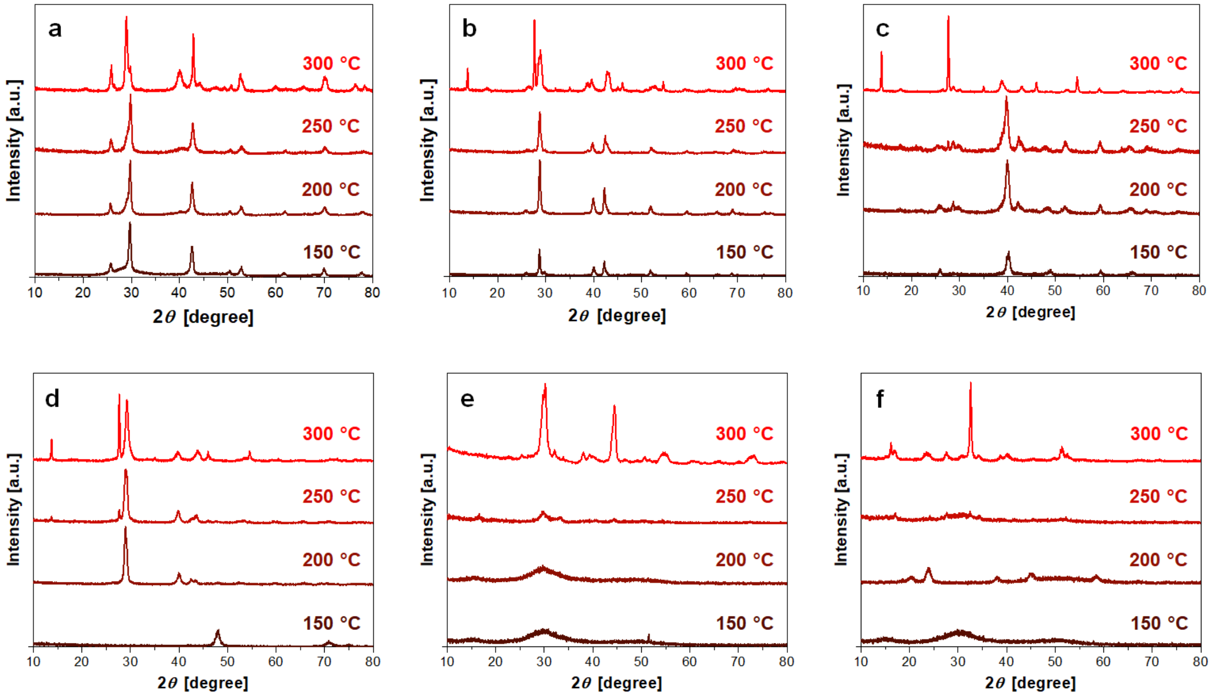

Supplementary Figure 7. X-ray diffraction spectra of $Ge_2Sb_2Se_xTe_{5-x}$ films annealed at different temperatures: (a) $Ge_2Sb_2Te_5$ (GST-225); (b) $Ge_2Sb_2Se_1Te_4$; (c) $Ge_2Sb_2Se_2Te_3$; (d) $Ge_2Sb_2Se_3Te_2$; (e) $Ge_2Sb_2Se_4Te_1$ (GSS4T1); and (f) $Ge_2Sb_2Se_5$.

Supplementary Figure 7 summarizes the X-ray diffraction (XRD) spectra of $Ge_2Sb_2Se_xTe_{5-x}$ films annealed at different temperatures in an argon protective atmosphere for 30 minutes. Our XRD analysis (not shown here) also shows that all as-deposited films are amorphous. For films with x = 0 to 4, the films first crystallize into a metastable phase followed by complete transition to the stable hexagonal structure, evidenced by the emergence of new diffraction peaks (e.g. the peak at 28 degrees for $Ge_2Sb_2Se_1Te_4$). For GST-225, the phase transition results in a slight shift of the main diffraction peak at 30 degrees to 29 degrees. These observations are consistent with prior reports in the material system[11]. The crystallization onset temperature T_c monotonically increases with Se substitution, whereas the transition temperature to the hexagonal phase remains between 250 ℃ to 300 ℃. As a result, the intermediate temperature regime for the metastable phase diminishes with increasing Se substitution.

We further compare XRD spectra computed from the crystal structures derived from DFT simulations with the experimental results to validate the DFT model. As an example, Supplementary Figure 8 compares the measured

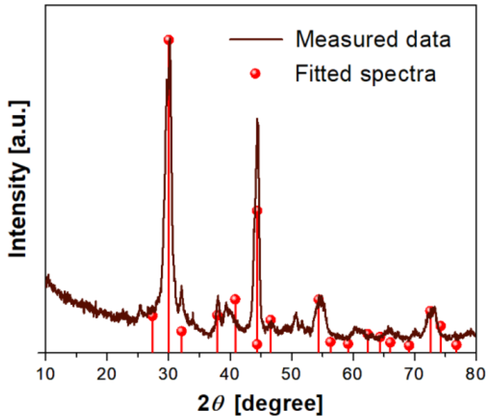

Supplementary Figure 8. Measured and fitted XRD spectra for hexagonal GSS4T1

and fitted XRD spectra for hexagonal GSS4T1 showing excellent agreement between the two. The fitting assumes the hexagonal structure predicted by our DFT modeling while allowing the unit cell to slightly dilate along its crystalline axes to obtain the best fit. The fitting yields lattice constants of a = 4.08 Å and c = 16.08 Å, very close to the DFT predicted values of a = 4.04 Å and c = 16.08 Å. The 1% distortion along the a-axis is likely caused by strains induced during crystallization of the film, as tensile strains in the order of 1% have been previously reported in thermally crystallized O-PCM films[12,13].

Supplementary Note 4: Crystallization behavior of $Ge_2Sb_2Se_xTe_{5-x}$

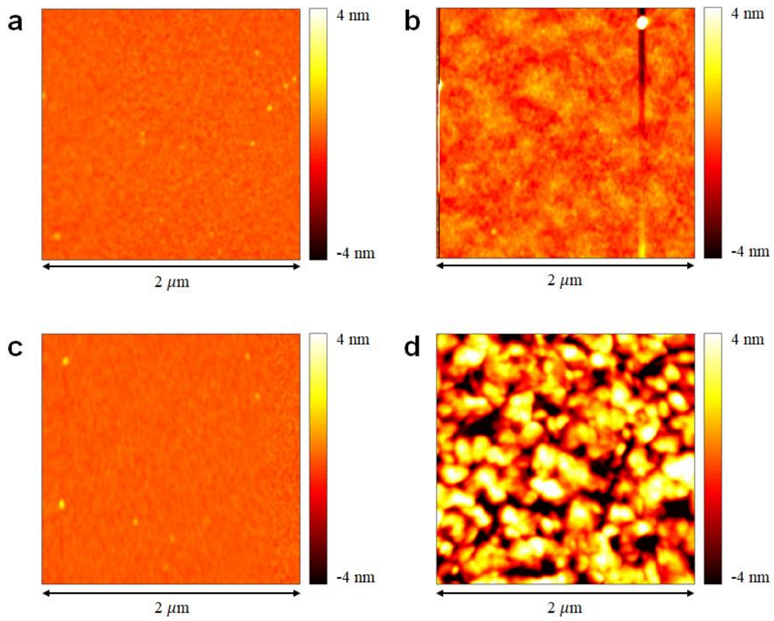

Supplementary Figure 9. Surface morphology of GST-225 and GSS4T1 films measured using AFM: (a) as-deposited GST-225; (b) thermally crystallized GST-225 (180 ℃, 30 min annealing); (c) as-deposited GSS4T1; (d) thermally crystallized GSS4T1 (300 ℃, 30 min annealing).

We investigated the crystallization behavior of $Ge_2Sb_2Se_xTe_{5-x}$ alloys through optical microscopy, selected area electron diffraction (SAED), and tapping mode atomic force microscopy (AFM). Supplementary Figure 9 shows the surface morphology of as-deposited and thermally crystallized GST-225 and GSS4T1 films. In both cases, the as-deposited films exhibit a small root-mean-square (RMS) roughness of < 0.2 nm, consistent with their amorphous nature. After crystallization, grain structures become apparent on the films, accompanied by a large increase of RMS roughness to 0.4 nm (GST-225) and 2.2 nm (GSS4T1). The average grain size in the thermally crystallized GSS4T1 film is 110 nm estimated from the AFM measurement. The presence of nanocrystalline grains in the thermally crystallized GSS4T1 sample is also independently proven through transmission electron microscopy (TEM) observation. Supplementary Figure 10a presents a high resolution TEM image of a GSS4T1 grain and the corresponding SAED pattern (inset) indicating the hexagonal structure.

In contrary, $Ge_2Sb_2Se_5$ exhibits a distinctively different crystallization behavior. The optical micrograph in Supplementary Figure 11 shows a crystalline grain in a $Ge_2Sb_2Se_5$ film deposited on Si, capped with a chemical vapor deposited thin SiO_2 layer (to prevent partial vaporization of constituents), and annealed at 300 ℃ for 30 minutes. The entire film is dotted with similar grain structures after annealing, and TEM imaging and SAED data (Supplementary Figure 10b) further confirm crystallinity of these grains with an orthorhombic structure. The

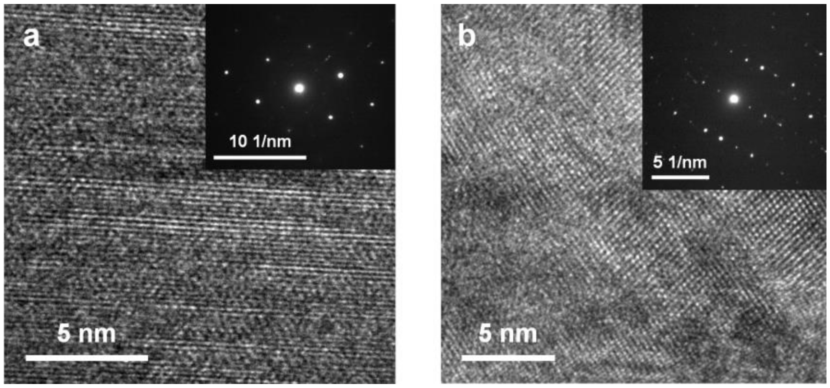

Supplementary Figure 10. TEM images and (inset) SAED patterns of thermally crystallized (a) GSS4T1; and (b) $Ge_2Sb_2Se_5$.

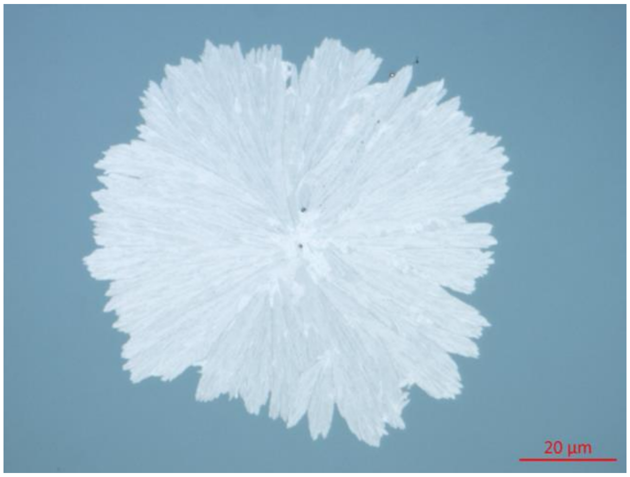

Supplementary Figure 11. Optical micrograph showing a crystalline grain in a $Ge_2Sb_2Se_5$ film annealed at 300 ℃ for 30 minutes

large grain size, coupled with the radial patterns inside the grains likely formed during outward growth of the grain, are strong indicators of a growth-dominated phase transition mode[14].

Supplementary Note 5: High-symmetry interfacial configurations in GSS4T1

The rotational stacking fault detected by TEM imaging in thermally crystallized GSS4T1 indicates the existence of many local minima within the relatively flat potential energy profile, which is ascribed to both the weak binding and symmetric characteristics at the Se-Te double layer (Supplementary Figure 12a and 12b). Since the interactions between atoms sharply decay with distance, the binding energy between adjacent atomic blocks is dictated by the contribution from the Se-Te double layer, while the remaining interactions can be viewed as perturbations. The binding strength between the Se and Te atoms depends on the pairwise distance as well as coordination number, and thus distinct interfacial configurations with 1/2/3-fold coordination likely exhibit similar binding strengths. Such degeneracy in combination with the high symmetry of interface lead to a large number of local minima with similar binding energies. Possible metastable configurations with representative rotational order between atomic blocks are schematically represented in Supplementary Figure 12c-i (c) global minimum with a single-crystal-like structure; (d) 60° rotation of (c); (e) translation of (c); (f) 60° rotation of (e); (g) 15° rotation of (c); (h) 30° rotation of (c); (i) 45° rotation of (c), wherein configurations (c-f) have the same double layer structure.

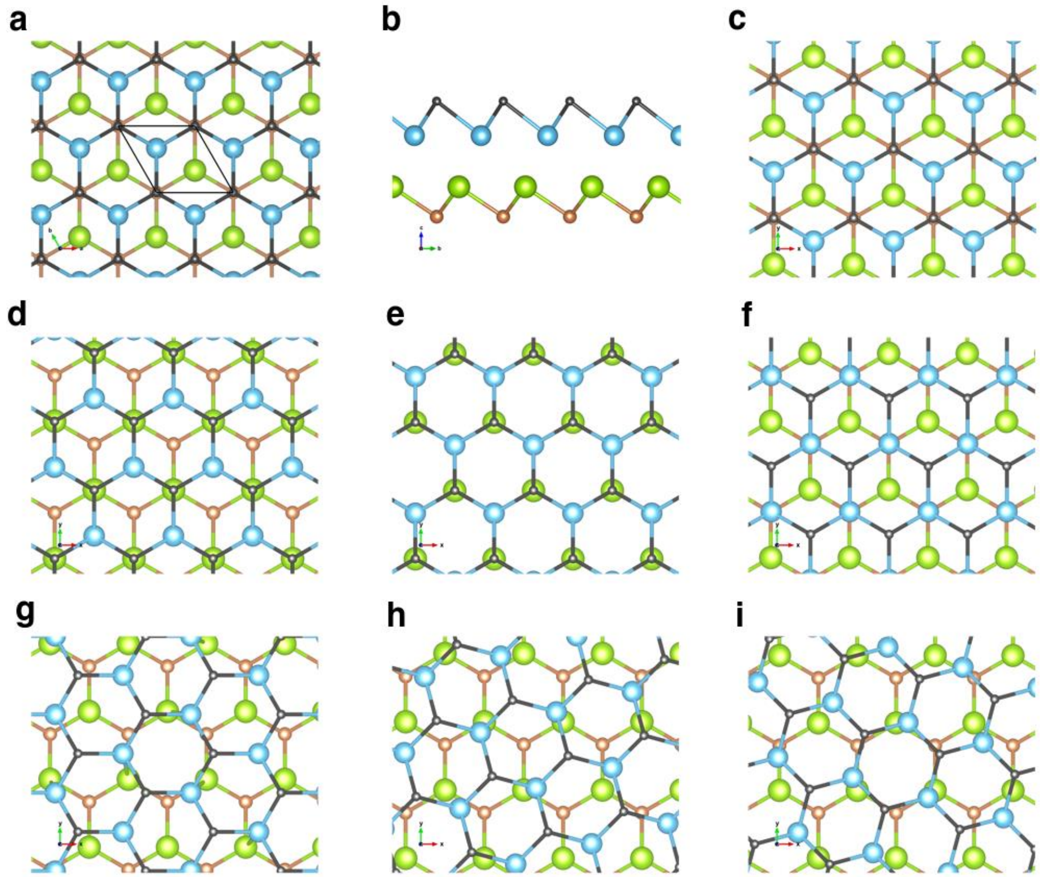

Supplementary Figure 12. Schematics of high-symmetry interfacial configurations: (a) top and (b) side views of the Te-Se double layer and its adjacent layers in hexagonal GSS4T1. (c-i) Possible metastable configurations with representative rotational order between atomic blocks (Te: blue, Se: green, Sb in bottom atomic block: brown, Sb in top atomic block: grey).

Supplementary Note 6: On non-metallic conduction behavior of GSS4T1

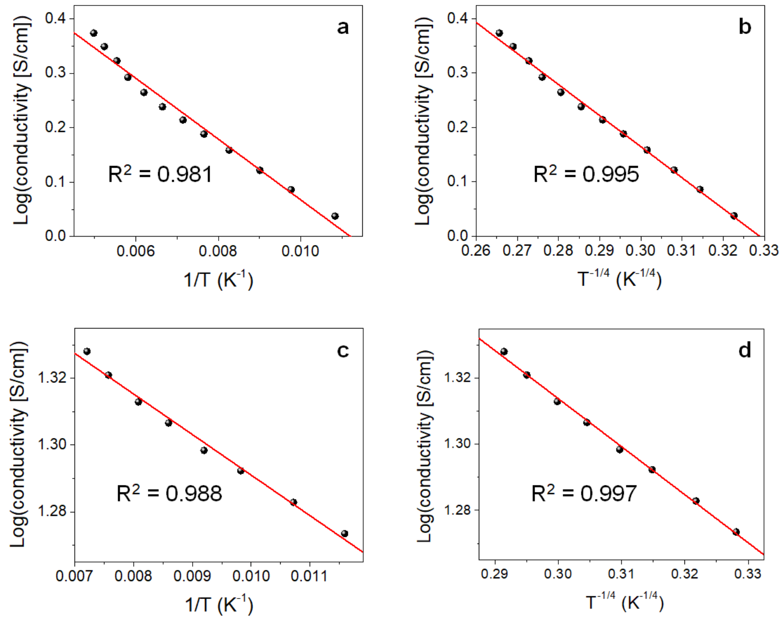

Supplementary Figure 13. Low-temperature conductivity of GSS4T1 annealed at (a, b) 265 ℃ and (c, d) 383 ℃ for 30 min fitted to (a, c) the classical Arrhenius law and (b, d) Mott's variable range hopping model. In both cases, the latter provides a better fit.

Following Siegrist *et al.*[15], here we distinguish the electrical conduction behavior of metals vs. non-metals by the sign of their temperature coefficient of resistivity (TCR): positive TCR is correlated to metallic behavior whereas negative TCR is a signature of non-metals. In GST-225 (as well as many other Ge-Sb-Te alloys of different stoichiometries), it has been observed that the conduction type of thermally crystallized material depends on the annealing temperature. At low annealing temperatures, the material tends to exhibit non-metallic characteristics (negative TCR) whereas metallic behavior (positive TCR) results when the sample is treated at elevated temperatures[15]. The conduction type transition is ascribed to vacancy ordering and reduced Anderson localization upon annealing at high temperatures[15,16]. The annealing temperature at which the TCR sign change occurs roughly coincides with the transition temperature from the cubic to the hexagonal phase, an observation consistent with other reports[17,18]. Following this definition, such metal-insulator transition (MIT) is absent in GSS4T1, even with high annealing temperatures when the material has completely transformed into the hexagonal phase (Fig. 3e). The result implies that in GSS4T1, the Fermi level always situates above the mobility edge and Anderson localization accounts for the low carrier mobility in c-GSS4T1. The conclusion is further supported by low-temperature conductivity measurement presented in Supplementary Figure 13 for c-GSS4T1 samples annealed at 265 ℃ and 383 ℃, respectively. In both cases, the temperature-dependent conductivity cannot be properly accounted for using the classical Arrhenius law, whereas Mott's variable range hopping model (the $T^{-1/4}$ law[19]) provides a satisfactory fit[20,21].

The nature of carrier localization (and the ensuing low mobility) in c-GSS4T1 is an open question and demands further study. Similar to its parent alloy GST-225, c-GSS4T1 exhibits large carrier concentrations far exceeding the critical carrier concentration to overcome electron correlation[15]. The critical carrier concentration for metallic transport can be evaluated by $n_c^{1/3} \cdot a_H = 0.26$, where a_H is the Bohr radius of the donor/acceptor state given by $a_H = 0.53\text{Å} \cdot \varepsilon_{st} \cdot m_e/m^*$, where ε_{st}, m_e, and m^* denote the static dielectric constant, electron mass, and effective mass of electron in the material. From DFT calculation, hexagonal GSS4T1 has an effective electron mass of 0.08 m_e, and a static dielectric constant of 44.8. This gives a critical carrier concentration of 6.7 $\times 10^{14}$ cm^{-3}, more than five orders of magnitude smaller than the carrier concentration of c-GSS4T1,

which exceeds 10^{20} cm^{-3}. According to the Mott's criterion, such high carrier concentrations should lead to strong metallic conduction behavior which contradicts our experimental results. In crystalline GST-225 where similar high carrier concentrations are observed, it has been suggested that Anderson localization of electrons resulting from disordered vacancies account for the insulator behavior of the alloy[15]. In GST-225 annealed at elevated temperatures, metallic behavior was observed which was attributed to ordering of the vacancies after high temperature treatment[16]. The absence of MIT in GSS4T1 likely point to either absence of such an ordering process, or additional localization mechanisms which cannot be removed via high-temperature annealing. The intrinsic structural disorder within the Te/Se double layer predicted by our DFT simulations (Supplementary Figure 3) can be one such possible mechanism, although further work is necessary to elucidate the nature of carrier localization in c-GSS4T1.

We also note that there are still remaining questions about the nature of electrical conduction in cubic GST-225[22]. The high carrier concentration extracted from Hall measurement results (Fig. 3c and other reports[17,23]) as well as absence of freeze-out at cryogenic temperatures[17] seem to indicate degeneracy in the material, i.e. the Fermi level should locate within a band. This conclusion, however, contradicts the negative TCR measured in cubic GST-225. In addition, there is experimental evidence from X-ray photoemission spectroscopy (XPS) suggesting that the Fermi level lies above valence band edge[24]. GSS4T1, whether in the cubic of hexagonal phase, exhibits the same anomaly as cubic GST-225 with a negative TCR and yet high hole concentrations.

Supplementary Note 7: Correlating material figure-of-merit with optical device performance

For the all-pass switch configuration shown in Fig. 5a, the ratio of transmitted (E_{output}) and incident (E_{input}) field in the bus waveguide is given by:

$$\frac{E_{output}}{E_{input}} = \frac{t - ae^{i\theta}}{1 - tae^{i\theta}}, \tag{VII.1}$$

where t is the coupling coefficient, θ denotes the single-pass phase shift imparted by the resonator, and a represents the inner circulation factor[25]. Considering a device loaded with a strip of O-PCM, the factor a accounts for the intrinsic waveguide loss, scattering loss at junctions between an unloaded waveguide and a O-PCM-covered waveguide, as well as material attenuation of the O-PCM. In our design, we introduced an adiabatic taper design to eliminate the junction loss. Therefore, a can be represented by:

$$a = a_0 \cdot a_{PCM} = a_0 \cdot e^{\frac{-2\pi\Delta L k_{eff}}{\lambda}}, \tag{VII.2}$$

where a_0 and a_{PCM} correspond to losses in the unloaded and O-PCM-loaded waveguide regions respectively, ΔL is the length of the O-PCM strip, and k_{eff} gives the modal extinction coefficient of the O-PCM-loaded waveguide. We further assume that when the O-PCM is at the amorphous state, the resonator is critically coupled to the bus waveguide at the working wavelength of λ = 1.55 μm. We then have:

$$\theta = 2m\pi, \qquad m = 1,2,3 \ldots, \tag{VII.3}$$

$$\text{and } t = a_0 \cdot e^{\frac{-2\pi\Delta L k_{eff(a)}}{\lambda}}. \tag{VII.4}$$

Here $k_{eff(a)}$ is the value of k_{eff} when the O-PCM is at its amorphous state.

When the O-PCM is transformed to the crystalline state, we have:

$$\frac{E_{output}}{E_{input}} = \frac{t - ae^{i\theta}}{1 - tae^{i\theta}}$$

$$= \frac{a_0 \cdot e^{\frac{-2\pi\Delta L k_{eff(a)}}{\lambda}} - a_0 \cdot e^{\frac{-2\pi\Delta L k_{eff(c)}}{\lambda}} e^{i\frac{2\pi\Delta L(n_{eff(c)} - n_{eff(a)})}{\lambda}}}{1 - a_0^2 \cdot e^{\frac{-2\pi\Delta L(k_{eff(a)} + k_{eff(c)})}{\lambda}} e^{i\frac{2\pi\Delta L(n_{eff(c)} - n_{eff(a)})}{\lambda}}}, \tag{VII.5}$$

and $k_{eff(c)}$ is the value of k_{eff} when the O-PCM is in its crystalline state.

Following the device structure we implemented in our experiments, the O-PCM strip is 450 nm wide and 50 nm thick, and sits on top of a 600-nm-wide, 400-nm-thick SiN waveguide. The refractive indices of GST-225 and GSS4T1 (based on our experimentally measured data) are shown below:

	GST-225	GSS4T1
Amorphous	4.69 + 0.19i	3.32 + 0.00017i
Crystalline	8.03 + 1.88i	5.08 + 0.35i

Here we introduce two additional figures-of-merit to assist in parameterizing the performance of an O-PCM integrated waveguide:

$$\mathrm{FOM}_n = \frac{n_{eff(c)} - n_{eff(a)}}{k_{eff(c)}} = \frac{\Gamma_c n_c - \Gamma_a n_a}{\Gamma_c k} \approx \frac{\Delta n}{k} \approx \frac{\Delta n}{\Delta k}, \quad \text{(VII.6)}$$

$$\mathrm{FOM}_k = \frac{k_{eff(c)} - k_{eff(a)}}{k_{eff(c)}}. \quad \text{(VII.7)}$$

Here Γ_c and Γ_a denote the modal confinement factors in the O-PCM strip at its crystalline and amorphous states, and n_c and n_a give the refractive index (real part) of the O-PCM at its crystalline and amorphous states, respectively. The approximation in Eq. VII.6 holds when the O-PCM strip thickness is sufficiently small compared to the waveguide core dimensions such that the perturbation due to phase change to the waveguide mode is small. In addition, optical loss in the amorphous phase is typically much smaller than that in the crystalline phase and is thus neglected. Under this assumption, $\Gamma_c \sim \Gamma_a$ and FOM_n reduces to the material FOM defined by Eq. 1 in the main text.

Equation X.5 then becomes:

$$\frac{E_{output}}{E_{input}} = \frac{e^{\frac{-2\pi\Delta L(k_{eff(c)}-k_{eff(a)})}{\lambda}} - e^{i\frac{2\pi\Delta L(n_{eff(c)}-n_{eff(a)})}{\lambda}}}{e^{\frac{4\pi\Delta L k_{eff(c)}}{\lambda}} - a_0^2 \cdot e^{\frac{2\pi\Delta L(k_{eff(c)}-k_{eff(a)})}{\lambda}} e^{i\frac{2\pi\Delta L(n_{eff(c)}-n_{eff(a)})}{\lambda}}} \cdot e^{\frac{2\pi\Delta L k_{eff(c)}}{\lambda}} \cdot a_0$$

$$= \frac{e^{\frac{-2\pi\Delta L \mathrm{FOM}_k \cdot k_{eff(c)}}{\lambda}} - e^{i\frac{2\pi\Delta L \mathrm{FOM}_n \cdot k_{eff(c)}}{\lambda}}}{e^{\frac{4\pi\Delta L k_{eff(c)}}{\lambda}} - a_0^2 \cdot e^{\frac{2\pi\Delta L \mathrm{FOM}_k \cdot k_{eff(c)}}{\lambda}} e^{i\frac{2\pi\Delta L \mathrm{FOM}_n \cdot k_{eff(c)}}{\lambda}}} \cdot e^{\frac{2\pi\Delta L k_{eff(c)}}{\lambda}} \cdot a_0 \quad \text{(VII.8)}$$

The effective indices of the O-PCM-loaded waveguide are derived from finite-difference modal simulations to be:

	GST-225	GSS4T1
Amorphous	2.28 + 0.10i	$1.80 + 3 \times 10^{-5}$i
Crystalline	4.78 + 1.74i	2.49 + 0.21i

FOMs of waveguides integrated with GST-225 and GSS4T1 are:

	GST-225	GSS4T1
FOM_n	1.43	2.81
FOM_k	0.9425	0.9998

When the O-PCM is switched to the crystalline phase, the device is at its OFF state. One of the most important performance metrics is insertion loss (IL), defined as:

$$\mathrm{IL} = -10 \cdot \log_{10}\left(\frac{I_{output}}{I_{input}}\right) = -10 \cdot \log_{10}\left(\left|\frac{E_{output}}{E_{input}}\right|^2\right). \quad \text{(VII.9)}$$

IL depends on the optical constants of O-PCM as well as its length ΔL. For a given material, a minimum IL (labeled as IL_{min}) can be obtained for a particular ΔL value. Below we examine how the material properties of O-PCM impact IL_{min}. Supplementary Figure 14 plot IL_{min} as functions of FOM_n and FOM_k for two a_0 values. In both cases, devices based on GSS4T1 claim significantly

improved performance compared to devices using GST-225, consistent with our experimental data. Here a_0 = 0.99 corresponds to waveguides (without O-PCM) with a moderate propagation loss: specific to our device configuration the corresponding SiN waveguide loss is 0.7 dB/cm. Supplementary Figure 14a indicates that in this case the device performance (IL_{min}) is largely dictated by FOM_n and hence the material FOM according to Eq. VII.6. The dependence of IL_{min} on FOM_k becomes pronounced only when the waveguide loss is extremely low (e.g, a_0 = 0.9999 or 0.7 dB/m as illustrated in Supplementary Figure 14b). The results are therefore in line with our statement that the optical performances of devices based on O-PCMs are largely determined by the material FOM.

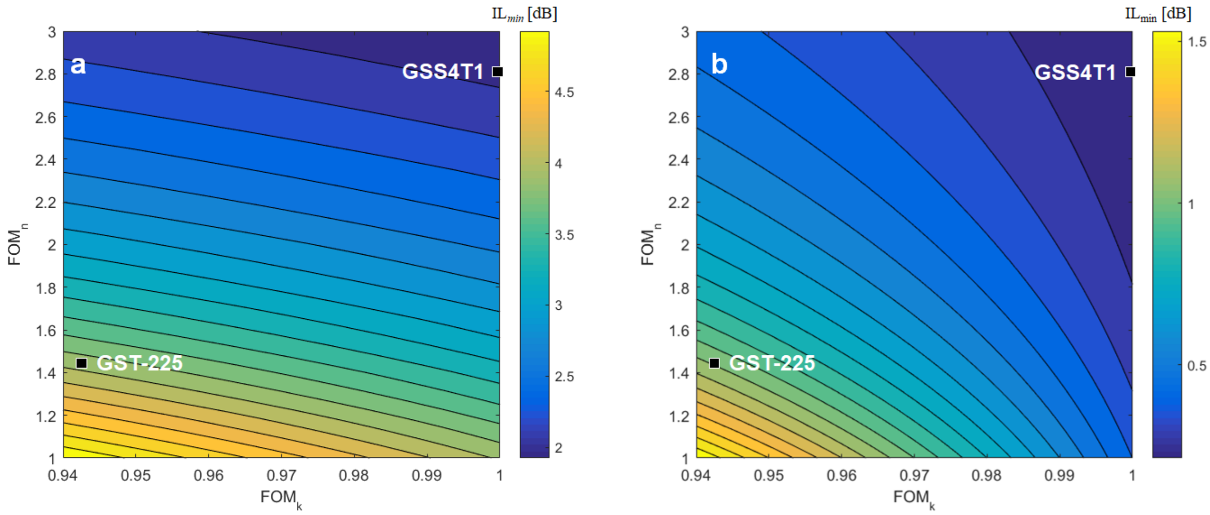

Supplementary Figure 14. Simulated dependence of device insertion loss on FOM_n and FOM_k for two unloaded waveguide loss cases: (a) a_0 = 0.99; (b) a_0 = 0.9999.

Supplementary Note 8: Cycling lifetime in electrical switching of GSS4T1

In order to test the long-term switching reliability, Supplementary Figure 15 shows the measured reflectance contrast between the amorphous and crystalline state on a single pixel over 1,000 cycles via electrothermal switching. The use of an inert Pt protective layer on the heater inhibits oxidation of Ti and improves the cycling lifetime of the device. A consistent contrast of ~ 21% (measured as the absolute reflectance difference between the two states of the devices) was maintained throughout the course of the test. No cracking or delamination was observed on the device after the 1,000 cycle switching test. As a comparison, we also modeled the optical reflectance contrast of the device based on the measured optical constants (Figs. 5a and 5b) of the materials via the transfer matrix method (TMM). The simulated contrast is indicated as the dotted line in the plot, showing excellent agreement with the experimental result. The slight differences likely result from deviations of the layer thicknesses from the design values.

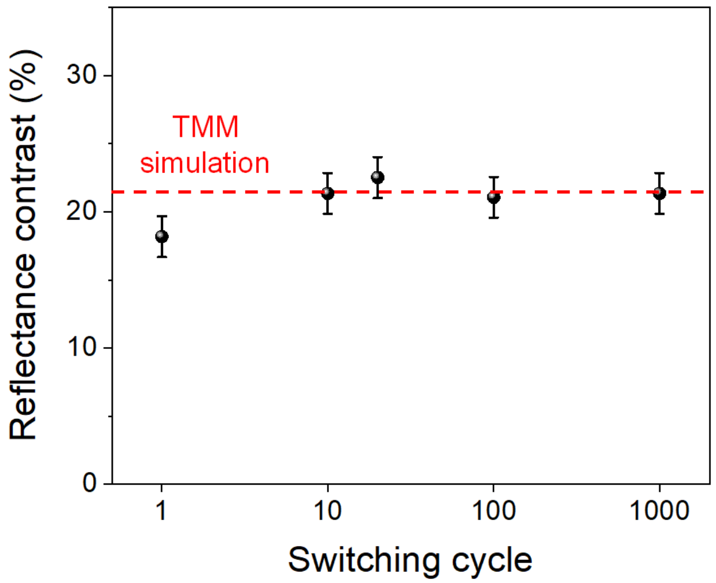

Supplementary Figure 15. Measured absolute optical reflectance contrast of an electrically switched GSS4T1 pixel over 1,000 cycles at a wavelength of 1500 nm.

Supplementary Note 9: Scaling pixel size and electrothermal switching energy

The amount of energy required to heat a volume of material to a specific temperature is quantified by the heat capacity $C = \rho V C_{sh}$, where ρ is the density, V is the volume, and C_{sh} is the specific heat of the material. In the case of the electrothermal heater design, the device heat capacity is a complex parameter defined by the phase change material, heater layer, interface layers, and the substrate. However, in general we should expect that the switching energy should scale with pixel sizes, since the entire heating volume has increased. Supplementary Figure 16 shows the experimental confirmation of the switching energy as a function of pixel sizes for both the crystallization and amorphization switching directions. The energy scaling with pixel sizes will serve an important function for application designs. For example, if low switching energies are required, the pixel size must be minimized as a result.

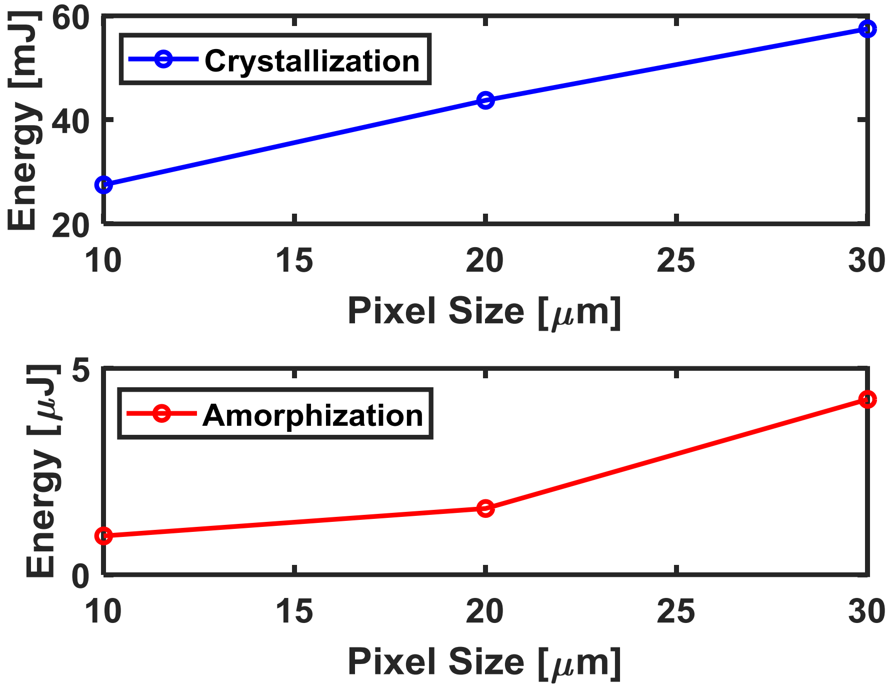

Supplementary Figure 16. Measured electrical switching energies for both crystallization and amorphization processes versus square pixel size.

Supplementary References

1 Svoboda, R. & Málek, J. Amorphous-to-crystalline transition in Te-doped $Ge_2Sb_2Se_5$ glass. *J. Therm. Anal. Calorim.* **117**, 1073-1083 (2014).

2 Matsunaga, T. *et al.* Structural analysis of GeSbTeSe phase-change materials. *Libr E\PCOS* **2009**, 99-104 (2009).

3 Sun, Z. M., Zhou, J. & Ahuja, R. Structure of phase change materials for data storage. *Physical Review Letters* **96**, doi:10.1103/PhysRevLett.96.055507 (2006).

4 Elliott, S. R. Chalcogenide Phase-Change Materials: Past and Future. *International Journal of Applied Glass Science* **6**, 15-18 (2015).

5 El-Din, K. S. a. E. A. M. a. F. S. T. a. A. S. a. S. M. Optical properties of amorphous chalcogenide thin films: The effect of Te isovalence substitution in the Ge-S-Se system. *Journal of Physics D: Applied Physics* **27**, 156 (1994).

6 Sun, Z., Zhou, J. & Ahuja, R. Structure of Phase Change Materials for Data Storage. *Phys. Rev. Lett.* **96**, 055507 (2006).

7 Hegedüs, J. & Elliott, S. R. Microscopic origin of the fast crystallization ability of Ge–Sb–Te phase-change memory materials. *Nat. Mater.* **7**, 399–405 (2008).

8 Lee, T. H. & Elliott, S. R. Ab Initio Computer Simulation of the Early Stages of Crystallization: Application to $Ge_2Sb_2Te_5$ Phase-Change Materials. *Phys. Rev. Lett.* **107**, 145702 (2011).

9 Ohara, K. *et al.* The Roles of the Ge-Te Core Network and the Sb-Te Pseudo Network During Rapid Nucleation-Dominated Crystallization of Amorphous $Ge_2Sb_2Te_5$. *Adv. Funct. Mater.* **22**, 2251–2257 (2012).

10 Matsunaga, T. *et al.* From local structure to nanosecond recrystallization dynamics in AgInSbTe phase-change materials. *Nat. Mater.* **10**, 129–134 (2011).

11 Koch, C. *et al.* Enhanced temperature stability and exceptionally high electrical contrast of selenium substituted $Ge_2Sb_2Te_5$ phase change materials. *RSC Advances* **7**, 17164-17172 (2017).

12 Nazeer, H., Bhaskaran, H., Woldering, L. A. & Abelmann, L. Young's modulus and residual stress of GeSbTe phase-change thin films. *Thin Solid Films* **592**, 69-75 (2015).

13 Pedersen, T. L. *et al.* Mechanical stresses upon crystallization in phase change materials. *Appl. Phys. Lett.* **79**, 3597-3599 (2001).

14 Burr, G. W. *et al.* Phase change memory technology. *Journal of Vacuum Science & Technology B* **28**, 223-262 (2010).

15 Siegrist, T. *et al.* Disorder-induced localization in crystalline phase-change materials. *Nature Materials* **10**, 202 (2011).

16 Zhang, W. *et al.* Role of vacancies in metal–insulator transitions of crystalline phase-change materials. *Nature Materials* **11**, 952 (2012).

17 Lee, B.-S. *et al.* Investigation of the optical and electronic properties of $Ge_2Sb_2Te_5$ phase change material in its amorphous, cubic, and hexagonal phases. *J. Appl. Phys.* **97**, 093509 (2005).

18 Friedrich, I., Weidenhof, V., Njoroge, W., Franz, P. & Wuttig, M. Structural transformations of $Ge_2Sb_2Te_5$ films studied by electrical resistance measurements. *J. Appl. Phys.* **87**, 4130-4134, doi:10.1063/1.373041 (2000).

19 Mott, N. F. Conduction in non-crystalline materials: III. Localized states in a pseudogap and near extremities of conduction and valence bands. *Philos. Mag.* **19**, 835-852 (1969).

20 Hu, J. *et al.* Studies on structural, electrical, and optical properties of Cu doped As–Se–Te chalcogenide glasses. *J. Appl. Phys.* **101**, 063520, doi:10.1063/1.2712162 (2007).

21 Hauser, J. & Kimerling, L. Electrical conduction in Si-implanted amorphous Si. *Physical Review B* **11**, 4043 (1975).

22 Raoux, S. & Wuttig, M. *Phase Change Materials: Science and Applications*. (Springer US, 2010).

23 Lyeo, H.-K. *et al.* Thermal conductivity of phase-change material $Ge_2Sb_2Te_5$. *Appl. Phys. Lett.* **89**, 151904, doi:10.1063/1.2359354 (2006).

24 Kim, J.-J. *et al.* Electronic structure of amorphous and crystalline $(GeTe)_{1-x}(Sb_2Te_3)_x$ investigated using hard x-ray photoemission spectroscopy. *Physical Review B* **76**, 115124, doi:10.1103/PhysRevB.76.115124 (2007).

25 Yariv, A. Universal relations for coupling of optical power between microresonators and dielectric waveguides. *Electron. Lett.* **36**, 321-322 (2000).
